# Mismatch Negativity (MMN) response studies in elderly subjects

**DOI:** 10.1016/S1808-8694(15)30545-0

**Published:** 2015-10-19

**Authors:** Gabriela Buranelli, Marcella Brito Barbosa, Cristiane Fregonesi Dutra Garcia, Sinésio Grace Duarte, Antônio Carlos Marangoni, Lucinda M. de F. Rodrigues Coelho, Ana Cláudia Mirândola Barbosa Reis, Myriam de Lima Isaac

**Affiliations:** 1 Speech and hearing therapist - University of Franca - Franca/SP; 2 Speech and hearing therapy - University of Franca - Franca/SP; 3 Public Health - USP - Ribeirão Preto/SP; PhD in Medical Sciences, Department of Otorhinolaryngology, Ophthalmology and Head and Neck Surgery- Medical School of Ribeirão Preto/SP - USP, Speech and hearing therapist. Professor of Speech and Hearing Therapy. University of Franca - Franca/SP; 4 Surgery - Medical School of Ribeirão Preto/SP - USP, MD. Neurologist. Professor at the Speech and Hearing Program - University of Franca - Franca/SP; 5 Bioengineering - Medical School of Ribeirão Preto/SP - USP / EE São Carlos. MSc in Solid State Physics - IFUSP - São Paulo/SP. PhD Student of Sciences - University of Franca - Franca/SP. Physicist. Professor of the University of Franca; 6 Bioengineering - Medical School of Ribeirão Preto/SP - USP / EE São Carlos. MSc in Solid State Physics - IFUSP - São Paulo/SP. PhD Student in Sciences - University of Franca - Franca/SP. Physicist. Professor of the University of Franca; 7 Human Communication Disorders - UNIFESP/SP. Speech and Hearing Therapist. Professor of Speech and Hearing Therapy - Department of Ophthalmology, Otorhinolaryngology and Head and Neck Surgery - Medical School of Ribeirão Preto/SP - USP; 8 Medical School of Ribeirão Preto/SP. Otorhinolaryngologist. Professor at the Department of Otorhinolaryngology, Ophthalmology and Head and Neck Surgery - Medical School of Ribeirão Preto/SP - USP

**Keywords:** attention, auditory cortex, memory, evoked potentials

## Abstract

Mismatch Negativity is an endogenous potential which reflects the processing of differences incurred in the acoustic stimulus.

**Aim:**

to characterize MMN responses in elderly subjects and compare with adult subjects.

**Materials and methods:**

prospective study involving 30 subjects, 15 men and 15 women, aged between 60 and 80 years and 11 months. Statistical test: Mann-Whitney. The subjects went through medical evaluation, threshold tonal audiometry, immittance tests, otoacoustic emissions and short and long latency auditory potentials (MMN).

**Results:**

mean latency was 161.33 ms (CZA2) and 148.67 ms (CZA1), in women; of 171 ms (CZA2) and 159.07 ms (CZA1), men. Mean amplitude was −2.753 μV (CZA2) and −2.177 μV (CZA1), women; −1.847 μV (CZA2) and −1.953 μV (CZA1), men. As to the right and left hemispheres, mean latency variable of 166 ms (CZA2) and 153.87 ms (CZA1); for the amplitude variable, mean value of −2.316 μV (CZA2) and −2.065 μV (CZA1).

**Conclusion:**

there is no statistically significant difference between the latency and amplitude when we compared males and females, right and left sides in the elderly and between chronologic ages between adults and elderly subjects.

## INTRODUCTION

The auditory system can be broken down into peripheral and central^1^. Threshold Tonal Audiometry (TTA) is the basis of any audiologic evaluation. Such tests establish the hearing thresholds and compare the values found with normal standards^2^. Hearing assessment by electrophysiological means aims at helping in the diagnostic and solution of hearing disorders^3^. Auditory Evoked Potentials (AEP) assess the neuroelectrical activity on the auditory pathway, from the auditory nerve all the way to the cerebral cortex, in response to a stimulus or acoustic event. They can be classified according to the latency in which they occur. Early AEP or short latency AEP happen in the first 10 milliseconds (ms), the medium latency potentials happen between 10 and 80ms and the late or long latency ones happen between 80 and 750ms^4^.

**Figure 1:**
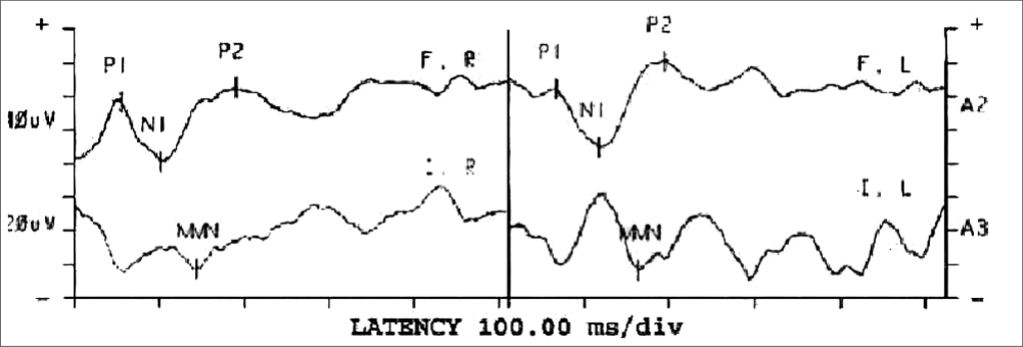
Results from the MMN test - ms - milliseconds P1/N1/P2 - Exogenous Potentials (Positive and Negative) F / I - Frequent / Infrequent R - Right; L - Left; MMN - Mismatch Negativity

The Brainstem Auditory Evoked Potentials (BAEP) arise from the auditory nerve and auditory pathways of the brainstem. The general BAEP format includes a series of seven positive waves. Wave V is the response with the highest value, for it is the larger and the least variable one, and it persists even at low intensities^3^, ^4^. Medium latency potentials are made up of a series of positive and negative waves. The first wave is the Na, the second is the Pa, afterwards we have the Nb, Pb and, sometimes Nc and Pc^5^. Currently, medium latency potentials have been seen as one of the most promising electrophysiologic tests, capable of assessing Central Nervous System dysfunctions^6^. Long latency potentials are established by the attention the individual pays to the sound stimulus. These potentials originate from the primary and secondary areas of the auditory cortex and are useful to study cognitive functions and attention. They can be broken down into exogenous and endogenous^4^, ^7^. The long latency potential called Mismatch Negativity - MMN - which is an endogenous potential, an automatic brain response which reflects the central processing of very subtle differences seen in the acoustic stimulus. This response is passive and does not require a behavioral response or attention^4^. The MMN is obtained by subtracting the wave of the infrequent stimuli from the wave of the frequent stimuli; then one obtains a third wave in which we can identify the presence of a potential of greater amplitude between the 100 and 250ms amplitudes (Figure 1).

Having in mind the growth in the elderly population and the difficulty to find MMN test parameters in Brazil, we had this interest in studying its characterization, in these subjects, for its results help investigate Central Auditory System (CAS) functions. Some studies^8^, ^9^ reported on the MMN response in normal adult subjects. Based on our findings, the present study compared the characteristics of the test in these different populations - adults and the elderly. The goal of this present study, therefore, is to characterize MMN response in the elderly, of both genders, with ages ranging between 60 and 80 years and 11 months; and to compare the MMN test characteristics in the adult and elderly populations.

## MATERIALS AND METHODS

**Figure 2:**
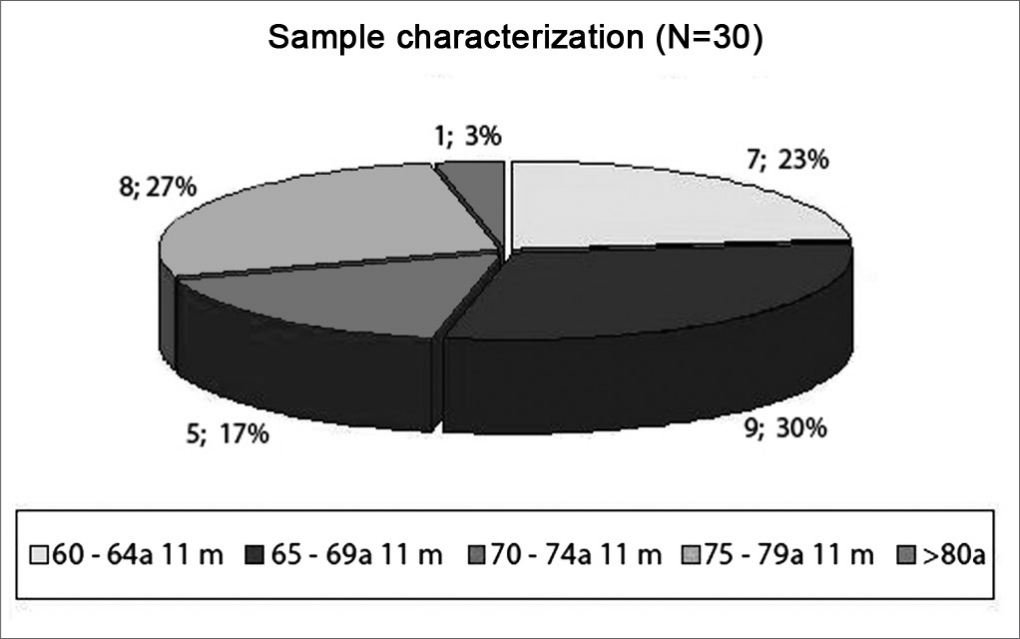
Study Sample Characterization (n=30) - N: number of subjects; a: years m: months

Prevalence study. Deductive method; descriptive, observational, cross-sectional, statistical, comparative and prospective. This research is focused on diagnostic purposes. The population in this study was made up of elderly subjects, with chronological age above that of 60 years, of both genders. The sample was made up of 15 men and 15 women, with ages ranging between 60 and 80 years and six months (Figure 2). The mean age for females was of 70 years and two months, and the minimum age was 60 years and five months and the maximum of 80 years and five months; for males, the mean age was 70 years; the minimum was 60 years and the maximum was 78 years and six months.

The dependent variables studied were the results from the MMN test and the independent variables were the CZA1 (left side), CZA2 (right side) leads; males and females; chronological age (adults and elderly). It is worth noticing that the data regarding the adults is present in two recent studies^8^, ^9^.

The equipment used were: HEINE mini 2000 Otoscope; (AC33 audiometer, TDH 39 phone), calibrated on October 18, 2007; middle ear analyzer (AZ-7), calibrated on October 18, 2007; ILO 292 OAE analyzer, version 5.0, Otodynamics LTDA, coupled to a conventional computer; Biologic version 5.70, model 317, two channels, coupled to a conventional computer; 29-inch Gradiente TV set; Philips DVD player; DVD (Movie) Green Alert.

Data collection procedures were carried out in the following stages: 1st stage: Approval from the Research Ethics Committee (approval # 0018/08); 2st stage: Free and Informed Consent signed by the participants or persons responsible for them; 3rd stage: neurological medical evaluation, in order to establish clinical diagnoses and obtain data on the history of the subjects studied; 4th stage: individual interview with the participants in order to collect data on their hearing and general health status; 5th stage: Inspection of the external auditory meatuses before the proper tests were carried out; 6th stage: TTA tests, immittance tests in order to find the tonal thresholds and check the middle ear and central auditory conditions; 7th stage: OAE and BAEP tests in order to study the outer hair cells and the central auditory pathways, considering: trace morphology, absolute latency of waves I, III and V and interpeak latencies of intervals I-III, III-V, I-V, as well as amplitude relationships of waves I and V10. As to the transitory OAEs, they were absent, and the Distortion Product OAE (DPOAE) in order to check for result compatibility; 8th stage: MMN testing, with the same equipment used to do the BAEP, however with the patient wide awake, without paying attention to the test. The patient had to watch the “Green Alert” movie, while the waves were recorded. We used a pure tone for the rare and frequent stimuli in performing the MMN with one channel for data collection. Lead placement followed the international 10/20 standard^12^ and the active lead (Cz) was placed on the center of the head, on the scalp, on input jack 1 of the pre-amplifier; the A1 and E2 Reference Leads (left and right ears, respectively), were placed on the earlobes, the tested ear was on input Jack 2 of channel 1 and the contralateral was not plugged to the preamplifier. The ground lead (Fpz) was placed on the forehead, near the hairline. After wave recording, the ones obtained by subtracting the rare stimuli by the frequent stimuli in order to identify the presence of endogenous MMN potentials, considered between 100 and 250ms, of greater negative amplitude in this interval.

The test parameters were based on some studies^4^, ^8^, ^9^, ^11^ with some adaptations for the present study, and we used the Tone Burst, with a gain of 15,000, intensity of 70 dBHL for rare and frequent stimuli, frequency 1,000Hz for the frequent stimulus and of 1,500Hz for the infrequent stimuli, 30.0 filter for the high frequencies and 1.0 for the low frequencies, 512 analysis time, supra-aural phone (TDH-39), rarefaction polarity, stimuli relation set on 5. For the sample, we used approximately 400 frequent stimuli (80%) and 100 infrequent stimuli (20%).

As to data analysis, in order to compare the genders, ear side and ages (adults and elderly) in terms of MMN amplitude and latency measures, we used the Mann-Whitney statistical test, because they are continuous values, measured in an interval scale of different groups, without any populational distribution of the variables^13^. The significance level (p value) was established on 5% (p=0.05).

## RESULTS

Table 1 shows the statistical data regarding the TTA test, for the subjects of both genders in the same sample, for the right and left ears.

**Table 1:** TTA value description according to gender.

Left ear	Women (n=15)	Men (n=15)	Right ear	Women (n=15)	Men (n=15)
Minimum	10 dBHL	10 dBHL	Minimum	10 dBHL	10 dBHL
Maximum	45 dBHL	90 dBHL	Maximum	45 dBHL	70 dBHL
Mean	26 dBHL	33,33 dBHL	Mean	26,33 dBHL	31,33 dBHL
Median	25 dBHL	25 dBHL	Median	25 dBHL	25 dBHL
Standard Deviation	10,036	22,414	Standard Deviation	9,904	16,198
p value 0.7084			p value 0,5330		

dBHL = deciBel Hearing Level

On Table 2 we describe the values regarding the MMN test latency variable, obtained from the assessment of both genders on the CZA1 and CZA2 leads. Table 3 describes the values related to the MMN amplitude, obtained from assessing both genders on leads CZA1 and CZA2. On Table 4 we describe the values related to the MMN amplitude and latency obtained on the assessment of the entire sample, from leads CZA1 and CZA2.

**Table 2:** MMN latency, CZA2 and CZA1 lead value description according to gender.

Right side (CZA_2_)	Women (N = 15)	Men (N = 14)	Left side (CZA_1_)	Women (N = 15)	Men (N = 15)
Minimum	108ms	102ms	Minimum	108ms	100ms
Maximum	240ms	250ms	Maximum	232ms	224ms
Mean	161,33ms	171ms	Mean	148,67ms	159,07ms
Median	152ms	164ms	Median	142ms	154ms
Standard Deviation	43,086	47,947	Standard Deviation	33,404	37,841
p value 0,5854			p value 0,3615		

ms = milliseconds

**Table 3:** Description of the amplitude values of the MMN, CZA2 and CZA1 leads, according to gender.

Right side (CZA_2_)	Women (N = 15)	Men (N = 14)	Left side (CZA_1_)	Women (N = 15)	Men (N = 15)
Minimum	-0,600μV	-0,290μV	Minimum	-0,070μV	-0,330μV
Maximum	-7,710μV	-5,720μV	Maximum	-3,960μV	-3,750μV
Mean	-2,753μV	-1,847μV	Mean	-2,177μV	-1,953μV
Median	-2,740μV	-1,135μV	Median	-2,280μV	-1,980μV
Standard deviation	1,762	1,817	Standard deviation	1,093	1,095
p value 0,1121			p value 0,6236		

μV = microVolts

**Table 4:** Description of MMN amplitude and latency values and those for CZA2 and CZA1 leads, of subjects of the same gender.

Latency (ms)	Right side (CZA_2_) N = 29	Left side (CZA1) N = 30	Amplitude (μV)	Right side (CZA_2_) N = 29	Left side (CZA_1_) N = 30
Minimum	102ms	100ms	Minimum	-0,290μV	-0,070μV
Maximum	250ms	232ms	Maximum	-7,710μV	-3,960μV
Mean	166ms	153,87ms	Mean	-2,316μV	-2,065μV
Median	156ms	150ms	Median	-1,990μV	-2,135μV
Standard deviation	44,941	35,467	Standard deviation	1,816	1,081
p value 0,35111			p value 0,9637		

ms = milliseconds; μV = microVolts

**Figure 3:**
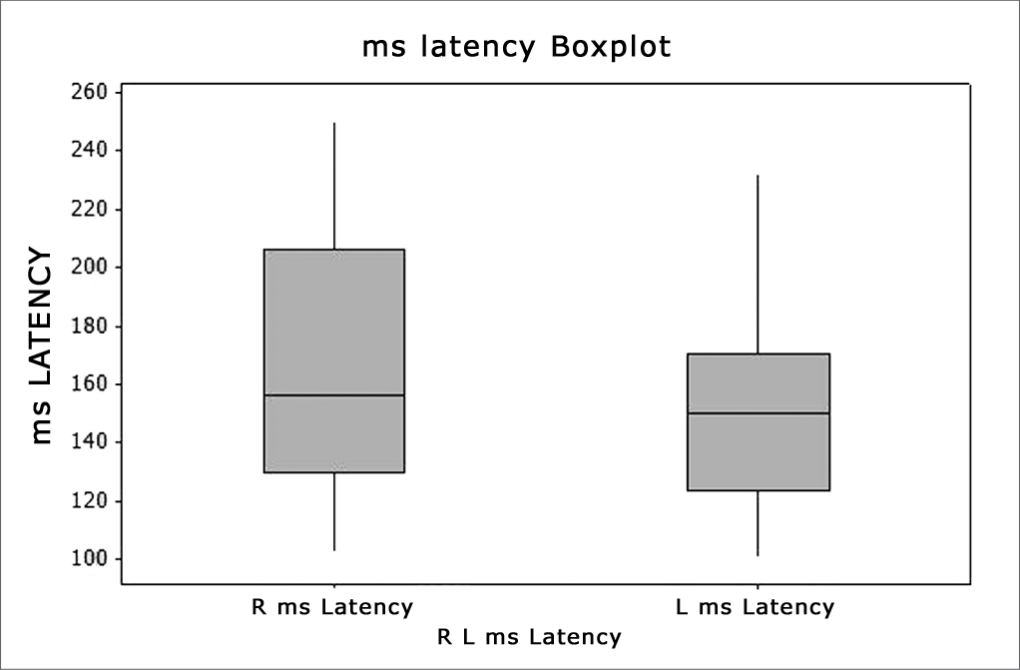
Boxplot - Latency Right and Left sides -ms - milliseconds R - Right (n=30 ears) L - Left (n=29 ears)

**Figure 4:**
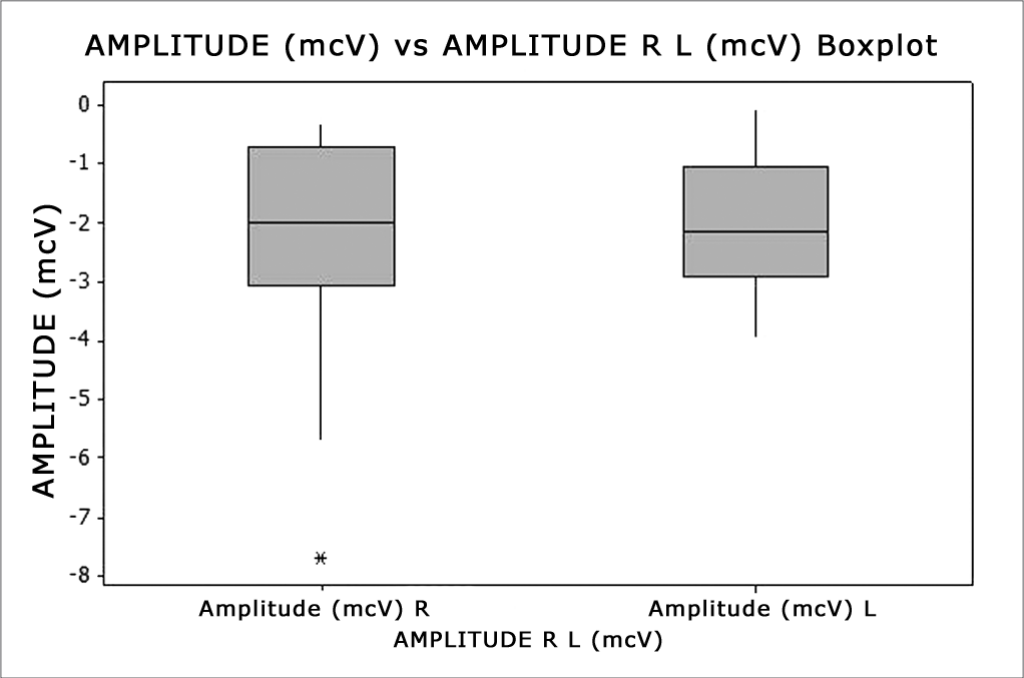
Boxplot - Amplitude Right and Left sides - mcV - microVolts R - Right (n=30 ears) L - Left (n=29 ears)

Figure 3, Figure 4 show the amplitude and latency values, of leads CZA2 and CZA1 of the MMN test of the entire sample (n=30), according to the statistical analysis, by means of the Boxplot graph.

In a second stage, we compared the groups of different chronological ages - adults and elderly. Data regarding the adults was obtained in the studies^8^, ^9^. Data regarding the elderly are the findings of the present study. Table 5, Table 6 describe the values regarding the latency and amplitude variables of the MMN exam, obtained on the assessment of adults and elderly from leads CZA1 and CZA2.

**Table 5:** Description of latency MMN, CZA2 andCZA1 leads, values in adults and children.

Right side (CZA_2_)	Adults (N = 12)	Elderly (N = 29)	Left side (CZA1)	Adults (N = 12)	Elderly (N = 30)
Minimum	150,20ms	102ms	Minimum	150,20ms	100ms
Maximum	245,20ms	250ms	Maximum	224,20ms	232ms
Mean	168,62ms	166ms	Mean	160,53ms	153,87ms
Median	154,70ms	156ms	Median	152,70ms	150ms
Standard deviation	29,352	44,941	Standard deviation	21,588	35,467
p value 0,6778			p value 0,3958		

ms = milliseconds

**Table 6:** MMN amplitude. CZA_2_ and CZA_1_ leads values from adults and children.

Right Side (CZA_2_)	Adults (N = 12)	Elderly (N = 29)	Left side (CZA_1_)	Adults (N = 12)	Elderly (N = 29)
Minimum	-0,500μV	-0,290μV	Minimum	-0,300μV	-0,070μV
Maximum	-9,450μV	-7,710μV	Maximum	-9,420μV	-3,960μV
Mean	-2,691μV	-2,316μV	Mean	-2,596μV	-2,065μV
Median	-2,180μV	-1,990μV	Median	-1,585μV	-2,135μV
Standard Deviation	2,596	1,816	Standard Deviation	2,535	1,081
p value 0,8748			p value 0,9778		

μV = microVolts

## DISCUSSION

The study sample was made up of thirty subjects, 15 women and 15 men (Figure 2). According to the statistical analysis, the minimum age for males and females was sixty years and five months; maximum age was 78 years and six months for males. Mean age for women was seventy years and two months and for males was seventy years. Comparing ages based on gender we noticed that there was no significant difference (p=0.9504). Life expectancy has increased and, for this reason, the population of the present study was chosen as the variable to be studied. Law 10.741/03 considers those above 60 years of age as elderly citizens^14^.

For data collection purposes, we carried out a basic hearing evaluation by means of the TTA, Speech Recognition Threshold (SRT), Speech Recognition Index (SRI) and immittance studies. According to Table 1, we did not observe significant statistical differences regarding right and left ears, females and males (p=0.5330; p=0.7084, respectively). The tonal threshold mean value for the right ear was 26.33 decibel Hearing Level (dBHL), for females and 31.33 dBHL for males. Minimum and maximum limits were 10 dBHL and 45 dBHL, right and left ears, for females; and in 10 dBHL and 90 dBHL, left ear for males. Based on this data, we diagnosed mild hearing loss, according to the mean values for both ears in both genders.

According to the literature studied, presbycusis is defined as the hearing loss stemming from an increase in chronological age^15^. According to other authors^16^, ^17^, the audiometric configuration of the elderly is usually a descending curve. For our analysis, the mean value of tonal thresholds was obtained according to the results of frequencies 500, 1 kilo (k), 2k and 3k Hertz (Hz). Therefore, the high frequencies, usually the ones impaired in the elderly population were not considered in this summation. That is why the level of hearing loss was non-significant in this study - contradictory to one study^18^, nonetheless matching another^19^. The tonal mean value by air conduction proposed 3 involves the thresholds of frequencies of 500, 1k and 2 kHz. We adapted our study to increase the frequency threshold to 3 kHz, in order to have a better evaluation of the entire cochlea, from base to apex. Results indicate a greater involvement of males - maximum and mean values according to the statistical analysis - not matching a study already carried out^20^. As far as logoaudiometry, SRT and SRI are concerned, the results from both ears on most of the subjects examined were compatible with the TTA. Except for the left ear of subject 29 - in whom we carried out the LDV, because of the profound hearing loss in this ear. The SRI was not compatible for both right and left ear, subject 19 and left ear for subject 24. We did not find data on the interview which would justify such result; therefore, we believe this may have happened because of the very advanced chronologic age, a hypothesis which was also mentioned in some studies^17^, ^20^. In the immittance test, we found 48.3% (29 ears) with type A tympanogram; 46.6% (28 ears), type As; 1.7% (1 ear) for curves Ad and C, besides one ear in which we could not properly seal the external acoustic meatus. Unmatching^20^ and matching^21^ findings, since the latter found in their sample a major occurrence of curve As among the elderly, which indicate a tympanic-ossicular stiffness, justified by the chronologic age. The percentage number of type A curves (48.3%), matched the type of sensorineural hearing loss seen in the sample subjects. As to the results of the stapedial reflex studies, we have observed that the present and/or absent responses matched the hearing threshold in the frequency tested. The DPOAE were studied and, when present, we completed the evaluation with the DPOAE. The goal was to check the outer hair cells of the inner ear. The present or absent responses matched the level of hearing in each ear, considering that the TOAE are present in people who can listen up to 30dBHL and DPOAE up to 50dBHL. In order to record the BAEP(s) we used adequate response criteria (35%, 21 ears), altered (48.3%, 29 ears) and wave I higher than wave V (16.7%, ten ears). In most of them, therefore, it presented an altered result (48.3%). This data matched that from some studies^7^, ^22^, ^23^, which assign such fact to the increase in chronological age.

According to the literature, the Long Latency Auditory Evoked Potential (LLAEP) happens between 80 and 750ms^4^, ^11^. In our study, we found MMN-LLAEP between 100 and 250ms.

Table 2 shows the results, according to the statistical treatment, regarding latency. For the right side (CZA2) the minimum value was 108ms for females and 102ms for males; the maximum value found was 240ms for females, and 250ms for males; the mean values was 161.33ms for females and 171ms for males. We did not find statistically significant differences (p=0.5854) for the right side when genders were compared. For the left side (CZA1), the minimum value was 108ms for females and 100ms, for men; the maximum value was 232ms for women and 224ms for men; the mean value was 148.67ms for women and 159.07ms for males. We did not find statistically significant difference (p=0.3615) for the left side, when the genders were compared.

Table 3 shows the results regarding amplitude. For the right side (CZA2), the minimum value was −0.600μV for women and −0.290μV for men; the maximum value was −7.710μV for women, and −5.720μV for men; the mean value was −2.753μV for women and −1.847μV for men. There was no statistically significant difference (p=0.1121) for the right side between the genders. For the left side (CZA1), the minimum value was −0.070μV for females and −0.330μV for males; the maximum value was −3.960μV for women and −3.750μV for men; the mean value was −2.177μV for women and −1.953μV for men. There was no statistically significant difference (p=0.6236) considering the left side for both genders.

We have also analyzed the latency and amplitude variables in the entire sample tested, a total of 29 subjects, 29 right ears and thirty left ears, since it was not possible to assess the right ear of subject # 24 because of the excessive number of artifacts, because of muscle pains reported by the patient.

Table 4 describes the latency in its different values: for the right ear the minimum was 102ms, a maximum value of 250ms and the mean value was 100ms; for the left side the maximum value was 232ms and the mean value was 153.87ms. We did not find statistically significant differences (p=0.3511) between the sides for the amplitude variable. Table 4 also describes the amplitude in its different values: for the right side, the minimum was 0.290μV, and the maximum was −7.710μV and the mean value was −2.316μV; for the left side the minimum was −0.070μV, maximum was −3.960μV and the mean value was −2.065μV. We did not find statistically significant difference (p=0.9637) between the sides regarding the amplitude variable. We can see the graph that shows these variables on Figure 3, Figure 4.

A study which did a LLAEP^24^ with senior citizens concluded that the exogenous potential latency (N1 and P2) was not affected in the presence of a hearing disorder. Such data was not discussed in our study. Other authors^25^, ^26^ advocate the idea that the perception of simultaneous sounds is made difficulty with the normal aging of the population, and this is the method of presentation of stimuli in the MMN test. In the literature studied we did not find MMN studies with elderly citizens showing latency and amplitude values.

Recent studies with adults^8^, ^9^, found mean latency values for the right side of 153.2ms and for the left side at 150.7ms for females; for the right side it was 184ms and for the left side it was 170.4ms for men in the MMN test. The mean amplitude for women was −3.548μV (right side) and −2.757μV (left side); and for men it was −1.867μV (right side) and −1.435μV (left side). They have reported statistically significant differences between the genders in both sides - lower latency for women; amplitude did not show statistically significant differences; nonetheless it was higher in women. In comparison to our findings, the mean latency was higher in the right side of the present study (161.33ms) and lower for the left side (148.67ms) for women. For men, latency was lower for both the right and left sides (171ms and 159.07ms, respectively). As far as the amplitude is concerned, our findings were lower for the right side (-2.753μV) and for the left side (-2.177μV) among women. For men, it was lower on the right side (-1.847μV) and on the left side (-1.953μV). Another study27 which evaluated subjects with severe and profound hearing loss (LLAEP - P300) reported mean latency of 371.3ms (right side) and 364.3ms (left side), at the age range between 25 and 45 years; and such data cannot be compared to our study, because it is a positive potential and not a negative one, like the MMN.

Some scholars^28^ observed that the latency is higher in adults and elderly patients when compared to their younger counterparts with the MMN. We compared the data associated with the elderly (present study) and adults (previous study^8^, ^9^), shown on Table 5, Table 6.

Table 5 shows the data associated with the latency variable. For the right side in adults (n=12 ears) the mean value was 168.62ms, the minimum value was 150.20ms and the maximum was 245.20ms. For the elderly (n=29 ears), the mean value was 166ms, the minimum value was 102ms and the maximum was 250ms. We did not find statistically significant differences (p=0.6778) among the ages, on the right side. For the left side, for adults (n=12 ears), the mean value was 160.53ms, the minimum value was 150.20ms and the maximum was 224.20ms. For the elderly (n=30 ears), the mean value was 153.87ms, the minimum value was 100ms and the maximum was 232ms. There was no statistically significant difference (p=0.3958) among the ages for the left side.

On Table 6 we see that the amplitude had a mean value of −2.691μV, the minimum value was −0.500μV and the maximum was −9.450μV, for adults (n=12 ears) for the right side. For the elderly (n=29 ears), in this same side the mean value was −2.316μV, the minimum value was −0.290μV and the maximum value was −7.710μV. There was no statistically significant difference (p=0.8748) among the ages considering the amplitude variable for the right side. The amplitude had a mean value of −2.596μV, the minimum value was −0.300μV and the maximum was −9.420μV for adults (n=12 ears), left side. For this same side, the elderly (n=30 ears) showed a mean value of −2.065μV, minimum value of −0.070μV and a maximum of −3.960μV. We did not notice statistically significant differences (p=0.6778) among the ages for the left side, considering the amplitude variable.

As it was found in the study26 which assessed subjects between 25 and 55 years and compared the healthy group with the group with Multiple Sclerosis (MS) and did not find statistically significant differences regarding the latency and amplitude variables, our study reports that the values of these variables in a group of higher chronological age (mean of seventy years for men and seventy years and two months for women), in subjects without significant clinical involvement (health status evaluated by the physician who participated in the study) and we also did not notice statistically significant differences in these variables, between the genders and, with one more data, between the left and right sides. Thus, as it happened in prior studies^8^, ^9^, we did notice, even without statistically significant differences, that the latency was lower and the amplitude was higher among women in the entire sample assessed in our study.

## CONCLUSION

There was no statistically significant difference when we compared the MMN characteristic variables, latency and amplitude, between elderly men and women comparing the right and left sides. There was no statistically significant difference between the chronological ages of adults and elderly.
